# Loneliness in online students with disabilities: qualitative investigation for experience, understanding and solutions

**DOI:** 10.1186/s41239-021-00301-x

**Published:** 2021-12-10

**Authors:** Yasuhiro Kotera, James Chircop, Lucy Hutchinson, Christine Rhodes, Pauline Green, Robert-Maxwell Jones, Greta Kaluzeviciute, Gulcan Garip

**Affiliations:** University of Derby, Kedleston Road, DE22 1GB Derby, UK

**Keywords:** Special needs education, Cultural and social implications, Distance education and online learning, Learning communities, Lifelong learning

## Abstract

Online learning has given access to education for diverse populations including students with disabilities. In our university, the ratio of students with disabilities is substantially higher in the online programmes than face-to-face. Online learning provides high accessibility though it can result in a lonely experience. Accordingly, this study aimed to appraise the first-hand experience and understanding of loneliness in online students with disabilities (OSWD), and to discuss possible solutions. Thematic analysis on semi-structured interviews attended by nine OSWD identified: ‘Self-paced study can reduce stigma but cause loneliness (Theme 1)’, ‘Loneliness and social difficulties relate to misunderstanding of disability (Theme 2)’, and ‘Activities, events and staff for informal socialisation are needed (Theme 3)’. As the demand for online learning is further expanded due to the current global pandemic, our findings will be helpful for online learning institutions worldwide to establish effective strategies to reduce loneliness in OSWD.

**Supplementary Information:**

The online version contains supplementary material available at 10.1186/s41239-021-00301-x.

## Introduction

The use of digital technology for education is increasing. Online learning has enjoyed steady growth in both popularity and prevalence (Kumar et al., 2017). Online learning has the potential to widen access and participation, particularly in higher education by eliminating the need for relocation and transportation, enabling financial savings and allowing for flexibility and self-paced study (Allen & Seaman, 2013; Kotera et al., 2019; Seale, 2013). For students with disabilities, online learning has provided more educational opportunities compared to campus-based study provisions with high accessibility (Kotera et al., 2019). This can be seen in the rise of students with a disability opting to study online, compared to traditional, on-campus university settings. While the rates of students with disabilities were similar at a national level in the UK (Higher Education Statistics Agency, 2020) and in the host university as a whole (15%; University of Derby, 2018), 40% of students studying in the online department of the host university disclosed a disability (Kotera et al., 2019), suggesting a preference for and the suitability of online study for students with a disability.

Opportunities include flexibility such as being able to structure learning around lifestyle requirements (e.g., treatment or care for disabilities), and engage with academic circles regardless of difficulties with vision, speech or mobility. Greater anonymity and opportunity for non-disclosure of disability are also regarded advantageous among OSWD (Seale, 2013). On the other hand, challenges may include the difficulties with online navigation including inadequate search functions, and with using multiple platforms to engage in learning activities (Habib et al., 2012) and a risk for marginalisation (Carr, 2011). There are only a few studies that have investigated challenges that OSWD experience in online learning, and the focus has primarily been on barriers in learning (Habib et al., 2012) and mental health (McManus et al., 2017). Despite that marginalisation and the mental health impacts were noted as a major challenge in OSWD (Carr, 2011; McManus et al., 2017), a sense of loneliness has not been investigated in depth.

### Impacts of loneliness on academic performance

Loneliness, an unpleasant psychological state resulting from deficiencies in an individual’s social network, has been reported as a risk factor for poorer academic engagement and success, compared to those who do not report feeling loneliness (Arkar et al., 2004; Lin & Huang, 2012). Loneliness can occur regardless of whether you are among others or not (Pinquart & Sörensen, 2001) and can be conceptualised as ‘social loneliness’, when a person does not feel integrated within a group, and ‘emotional loneliness’, the emotions experienced following the loss of a loved one (e.g., death or divorce; Weiss, 1973). In the context of loneliness experienced by online students with disabilities (OSWD), this study focused on social loneliness. Because humans are inherently social beings (Heinrich & Gullone, 2006), loneliness can significantly hinder our wellbeing (Diehl et al., 2018). Indeed, previous research has identified that feelings of loneliness were related to poor physical health, depression, and mental distress (Arkar et al., 2004; Wei et al., 2005; Wright et al., 2014). Relatedly, feelings of loneliness can also negatively impact academic performance and achievement (Benner, 2011), and has been linked as an underlying factor of dropout from university (Ali & Gregg Kohun, 2007; Kelly et al., 2007; Vayre & Vonthron, 2019). Understanding these feelings in OSWD can contribute towards improving their loneliness in online learning. Accordingly, this study aims to investigate whether online learning leads to loneliness in a range of disabilities.

Loneliness is considered to play a significant part in the increasing prevalence rates of mental illness and low well-being among students, and contribute to the rising demand for support services (Thorley, 2017). The rise in help-seeking from university counselling services is concerning (Novotney, 2014), as there tends to be limited resources to meet the students’ needs for this support. McIntyre et al. (2018) found that loneliness was the strongest predictor of academic distress, which was the strongest predictor for academic outcome in over 1000 UK undergraduates. These findings about loneliness are in line with the results of other studies in the UK (Richardson et al., 2017), Belgium (Diep et al., 2017) and the USA (Hefner & Eisenberg, 2009).

Though Ozaydın Ozkara and Cakir (2018) reported that interactive activities reduced students’ perception of loneliness among online students, loneliness among OSWD has not been studied in as much depth compared to students without disabilities. OSWD reported that the control they had over their studies and the personal touch (i.e., supportive interaction with university staff and peers) were especially important for them to succeed in online learning. On the other hand, they also reported challenges in socialising, and occasionally experienced a great amount of loneliness studying online (Kotera et al., 2019). Though previous research identified that a sense of loneliness had a detrimental impact in continuing online learning (Blau et al., 2020), how and the nature of loneliness OSWD experience has not been investigated in depth. This is particularly important in today’s educational environment: more and more students have reported increased levels of loneliness during the time of the coronavirus disease 2019 (COVID-19) pandemic (Kabashkin et al., 2021; No Isolation, 2020; O’Neill et al., 2020). Accordingly, the present study aimed to investigate OSWD’s first-hand experience of loneliness and discuss possible solutions.

### Study aims

This study aims to appraise the experiences and understanding of loneliness in online learning among students with disabilities (SWD), and to discuss possible solutions to help them cope with loneliness in their academic journeys. To help achieve this aim, three research questions were established;

RQ1: What are the experiences of online learning and loneliness in OSWD? (Experience.)

RQ2: How do they make sense of their feelings of loneliness? (Understanding.)

RQ3: What would help to reduce loneliness in online learning? (Future support.)

RQ1 explores OSWD’s subjective, first-hand experience of loneliness in online learning, whereas RQ2 asks about more objective, cognitive comprehension of loneliness among OSWD informing the knowledge of loneliness. RQ3 discusses the future support universities can offer to reduce loneliness in this student group.

## Materials and methods

### Research design

Online, semi-structured interviews with nine participants were conducted to understand the experiences and perceptions of OSWDs’ experiences of loneliness and social connectedness. Because our interviews involved questions about potentially sensitive topics, which would require attention to the way the participants make comments (Bryman, 1988), we employed videocall interviews. This study was reported following the consolidated criteria for reporting qualitative studies (Tong et al., 2007).

### Online learning environment

Participants engaged with an asynchronous virtual learning environment with multimodal contents, discussion forums, personal journals and announcements (Wang & Huang, 2018). Additionally, live webinars were offered three to five times in each module, providing synchronous learning using group videocalls. All webinars were recorded for those who could not participate.

### Participants

To be eligible, participants were a minimum of 18 years of age, declared a disability, and had a minimum of one year experience of learning online. Of the 19 students who agreed to participate, nine were interviewed by the researchers (eight women and one man; Age range = 22–56, M = 34.9, SD = 12.9 years; four psychology students, two healthcare students, and one environment, computing and education student each; six postgraduate and three undergraduate students; seven UK students and two international students; Table 1). Reasons for withdrawal were not asked following the ethical guidelines, however the ten students who had agreed but did not participate noted changes in their personal life including health and work issues. All of the nine students presented medical evidence of their disabilities, and eight of them were receiving the university’s disability support plan. Their declared disabilities were Chronic Fatigue Syndrome (*n* = 2), Dyspraxia (*n* = 2), ADHD, Anxiety Disorder, Arthritis, Asperger’s Syndrome, Bipolar, Chronic Allergies, Chronic Pain, Crohn's Disease, Dyslexia, Endometriosis, Mobility, Multiple Sclerosis, Muscular Dystrophy, and Myalgic Encephalomyelitis (each *n* = 1). All participants were full-time employees, as well as part-time online students.

**Table 1:** Participant list

Participant	Gender	Age	Programme	Disability	Residence
1	F	34	PG Education	Mobility, Bipolar	UK
2	F	23	UG Computing	ADHD, Asperger’s Syndrome, Chronic Fatigue Syndrome, Myalgic Encephalomyelitis	UK
3	F	25	PG Environment	Anxiety Disorder	UK
4	M	56	PG Healthcare	Dyspraxia	Other Europe
5	F	56	UG Healthcare	Dyslexia, Dyspraxia	UK
6	F	22	PG Psychology	Muscular Dystrophy	UK
7	F	31	PG Psychology	Arthritis, Endometriosis, Crohn's Disease	
8	F	31	PG Psychology	Multiple Sclerosis	UK
9	F	36	UG Psychology	Chronic Allergies, Chronic Pain, Chronic Fatigue Syndrome	Other Europe

*PG*  postgraduate, *UG*  undergraduate

Demographic data of our purposive sample of nine students covered diverse characteristics (i.e., programme of study, type of disability, residence, gender and age). All researchers were based in the UK. The nine interviews reached a point of saturation: the researchers agreed that (a) the data collected were adequate and meaningful to answer the research questions, and (b) more interviews would not yield any additional meaning to the overall story (Braun & Clarke, 2013).

### Procedure and analysis

Research information was disseminated through the announcement pages of the online learning environment by the programme leaders. Students who were interested in participating in the study submitted an online participation form, then were contacted by one of the researchers to arrange an hour-long videocall interview. To collect authentic responses from students, researchers who were not associated with the student’s programme were chosen as the interviewer. Authors JC, LH, CR, PG, and RMJ, all of whom were registered healthcare professionals and familiar with client’s safety and active listening skills, interviewed participants. Interviewers conducted interviews from the university. Participants were asked to inform their whereabouts of the interview to someone who could access the location of the interview. Pertinent information including all questions were provided beforehand to participants. Ethical approval for this study was granted by the university’s research ethics committee.

Previous research suggested that many SWD felt some degree of anxiety talking about issues related to their disabilities (Kotera et al., 2019). Acknowledging this limitation, we used a semi–structured interview guide that, on one hand, focused on participants’ experiences of loneliness in the context of online learning, but on the other, enabled them to tell their story as *individuals* (e.g., different attitudes toward socialising, instances where participants felt lonely in their modules, etc.). Again, acknowledging the boundaries of physical separation in online communications, we sought to maintain our videocall interviews as a natural form of communication by giving participants time to respond to questions (Burns, 2010). This provided a personal touch that E-interviews tend to lack (Bampton & Cowton, 2002).

Each interview explored how OSWD experience loneliness and social connectedness in the context of online learning; how they experience online learning and loneliness (RQ1); how they make sense of loneliness (RQ2); and what, in the students’ view, can be done to reduce loneliness in online learning (RQ3) (Additional file 1 for the interview schedule). During the interviews, researchers sought to use appropriate language, identify some of the digital ‘tacit signs’ and make participants feel comfortable (Woods, 2011) (e.g., when participants make interesting comparisons or need reassurance about their digital presence [a desire to show one’s online learning engagement and availability for communication; Gregory & Bannister-Tyrrell, 2017]). Interviews were audio-recorded and transcribed, then the accuracy of transcription was confirmed by the interviewers.

**Fig. 1:**
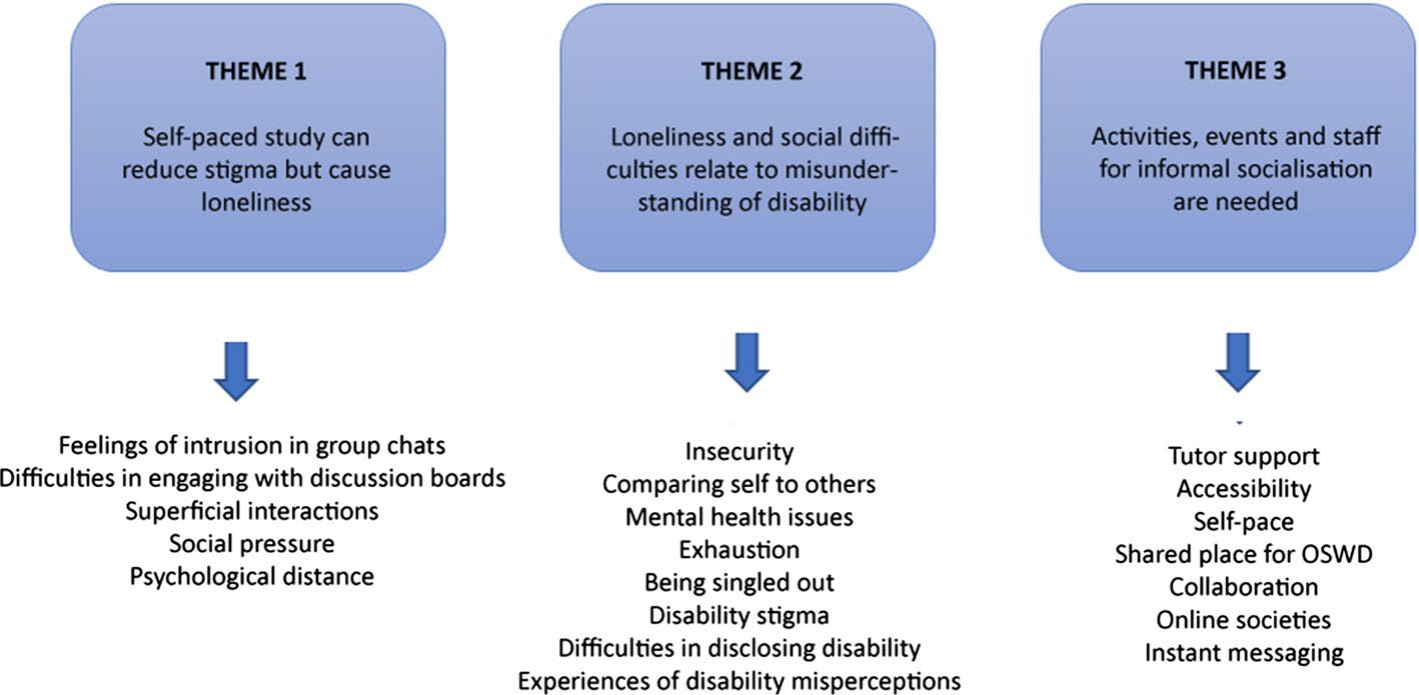
Organising codes into themes via mind map

Thematic analysis was used to analyse the interview data. We used Braun and Clarke’s (2006) guidelines on conducting a reflective thematic analysis, from a social constructivist epistemological framework. In order to determine the relevant themes in the interview data, we conducted thematic analysis in the following order: (i) Familiarisation, (ii) Generating initial codes, (iii) Searching for themes, (iv) Reviewing themes, and (v) Defining and naming themes (details on each process are available below) (Braun & Clarke, 2006). An investigator triangle (Hales, 2010) was established for research coherency: a pedagogic researcher who specialised in online learning and another researcher who specialised in student disabilities reviewed the data extracts associated with each theme identified by the co-researchers; both researchers agreed with all themes. Thus, our thematic analysis comprised of (Braun & Clarke, 2013):

1. *Familiarisation*
  Interview data was read repeatedly to establish initial interpretations and patterns (Bird, 2005).
2. *Generating initial codes*
  Coding was conducted to structure the interview data (Braun & Clarke, 2013; Tuckett, 2005). Codes are defined as data features that are interesting to the researcher and have a clear connection to the studied phenomenon (Boyatzis, 1998). It is important to note that the codes identified at this stage are not the same as themes (units of analysis), as the latter are generally much broader. Our coding was ‘theory–driven’ (Braun & Clarke, 2013) in the sense that we approached the data with three main research questions in mind.
  The twenty codes were: insecurity, mental health issues, comparing self to others, feelings of intrusion in group chats, social pressure, accessibility, self–pace, online societies, collaboration, instant messaging, disability stigma, difficulties in disclosing disability, superficial interactions, psychological distance, exhaustion, tutor support, difficulties in engaging with discussion boards, shared place for SWD, being singled out, and experiences of disability misperceptions.
3. *Searching for themes*
  Codes were categorised into potential themes by re-focusing data analysis at a broader level. Using mind map process (Braun & Clarke, 2006), each code was attached to a theme-pile (Fig. 1). The twenty codes were categorised into three themes: (i) Self-paced study can reduce stigma but cause loneliness, (ii) Loneliness and social difficulties relate to disability, and (iii) Activities, events and staff for informal dialogues. No codes were identified as an outlier.
4. *Reviewing themes*
  Data was reviewed in accordance with Patton's (1990) dual criteria for judging internal and external heterogeneity in order to establish thematic coherence and distinctions. Codes were re-read alongside the collated exacts to further refine the differences between the three themes (Braun & Clarke, 2013). The data were organised into three types: (i) tangible, affective experiences of loneliness in the university’s online learning space; (ii) perceptions toward as well as experiences of online socialising with a disability; and (iii) concrete ideas for the improvement of studying and social activities in digital learning environments. The theme ‘Self-paced study can reduce stigma but cause loneliness’ relates to participants’ experiences of loneliness (corresponding to RQ1); the theme ‘Loneliness and social difficulties relate to disability’ was linked to participants’ understanding of loneliness (RQ2); and finally, the theme ‘Activities, events and staff for informal dialogues’ conveyed participants’ ideas and wishes for improvements in online learning (RQ3).
5. *Defining and naming themes*

At this stage, we returned to collated data extracts to refine each theme and ensure an internally consistent account with accompanying narrative (Braun & Clarke, 2013).

Lastly, during the revision of the manuscript, the wording of Themes 2 and 3 were adjusted for accuracy and consistency: Theme 2 ‘Loneliness and social difficulties relate to misunderstanding of disability’, and Theme 3 ‘Activities, events and staff for informal socialisation are needed’.

## Results

Results were organised to respond to our research questions focussing on experiences (RQ1), understanding (RQ2), and future support (RQ3) of OSWD. The data extracts that comprised Theme 1 ‘Self-paced asynchronous study can reduce stigma but cause loneliness (RQ1)’ showed that many OSWD recognised the self-paced nature of online learning as an advantage: OSWD would not experience social anxiety because their disabilities are less visible than face-to-face settings. At the same time, however they noted that the self-paced asynchronous mode of study can cause loneliness. The extracts from Theme 2 ‘Loneliness and social difficulties relate to misunderstanding of disability’ demonstrated that loneliness and difficulties in their online social life were uniquely associated with their disabilities (e.g., misunderstanding of disabilities). The extracts from Theme 3 ‘Activities, events and staff for informal socialisation are needed’ highlighted the OSWD’s needs for non-formalised communications with staff and peers to reduce the sense of loneliness and increase social connectedness to their learning community. Participants’ comments were presented to clarify what each theme means, relating to all codes identified.

### Theme 1: self-paced study can reduce stigma but cause loneliness (RQ1: experience)

Participants related loneliness with the self-paced study of online learning. These students did not experience a high level of loneliness because they did not have high expectation for social connection in online learning in the first place.

Participant 7: I didn’t expect it to be social and don’t use online learning for socialisation.

Participant 6: I didn’t expect online learning to be social … I have one WhatsApp group with two other peers. But I don’t generally socialise [in] online learning.

These comments may highlight that online learning can help OSWD circumvent social anxiety associated with their disabilities (Kotera et al., 2019). Flexibility and self-paced nature of online learning allows students to have more control over their studies than face-to-face: online students can socialise (or not) and study at a pace that is comfortable and compatible with other aspects of their lives (Garip et al., 2020). Our participants noted the advantage of this self-paced, lone mode of study in relation to disabilities: not seeing staff and peers in person would reduce a sense of stigma associated with disabilities as those differences are less visible in online learning.

Participant 1: Online learning is great as it allows me as someone with a physical disability (wheelchair) to access higher education easily and comfortably. It is also great that I can gain insight from people that I ordinarily would not meet.

Participant 2: I think there is perhaps less stigma towards disabilities in the online classroom as it is harder to recognise if somebody suffers from a disability, especially if it is a physical one.

Although participating OSWD did not expect social connection and noted an advantage of online learning relating to reduction of stigma about disabilities, all participants consistently reported experiencing episodes of loneliness. Despite that participants demonstrated a generally good engagement with their online learning, some of them shared loneliness experienced in online learning and in their previous face-to-face learning experience.

Participant 1: When others are having discussions in the boards and I give my response, it feels like I am intruding and sometimes people will not respond to my posts. I also felt lonely when I [see] conversations between people who had obviously formed a bond in previous modules and knew things about each other.

This participant connected these feelings to her experience in the earlier face-to-face education.

Participant 1: It stems from when I was younger. When I first became disabled, I was taken out of school as it wasn’t accessible ... I only had a tutor once a week at home .... I was very isolated, and it was as though I was a burden and different to others.

The participant described a strong level of loneliness in face-to-face education and online learning.

Furthermore, other participants reported that their experiences of loneliness were often impacted by social (e.g., different student groups), technological (e.g., inability to participate in certain chat rooms) and/or temporal (e.g., feeling more anxious and lonelier toward the end of term or closer to deadlines) factors. Across these data extracts, students frequently noted that they prefer elements of face-to-face interaction to digital communications because of the depth of interaction.

Participant 8: I try to read everything on Blackboard and be involved in all the discussions but again it’s quite different from writing something down and being able to talk to someone. ... The loss of contact is the most important one. [There] is a feeling of [being] misunderstood because you cannot hear [students and teachers] when you write something down.

Participants highlight the importance of personal touch in online learning to counter their sense of loneliness. Without such contact, online learning can cause feelings of loneliness among OSWD (Boston & Ice, 2011; Ozaydın Ozkara & Cakir, 2018).

### Theme 2: loneliness and social difficulties relate to misunderstanding of disability (RQ2: understanding)

Most participants (*n* = 7) thought that having a disability has an immense impact on experiencing loneliness and difficulties in socialising with others when studying online.

Participant 4: Your disability might mean that you can’t sit for long. It might mean that you can’t read on the screen long. It might mean you can’t take in a whole lot of information quickly. You kind of get nearly left behind, and I think you’re at a greater disadvantage if you have a disability.

Participant 1: I think that it can sometimes be daunting to participate in activities with others and it may be difficult to socialise in other ways especially if someone has disabilities.

It is noteworthy that participants who did not think that their experiences of loneliness are impacted by their disability still conveyed disability-related worries in online learning.

Participant 8: Sometimes my condition is very bad, so [I] cannot be active on the forum. I do worry how I may come across to other students as not being active in the forum.

These comments may illustrate a sense of alienation, one component of loneliness (Hawkley & Cacioppo, 2010). Relatedly, participants reported insecurity when participating in peer discussions.

Participant 1: I often feel that if I participate, my thoughts and opinions will be ‘wrong’. It makes me feel intimidated that there are all of these amazingly bright people on the boards and then [there’s] me.

Participant 4: Everyone else is engaging in this conversation and you’re thinking ‘what are they talking about’ or ‘I couldn’t say anything, no, because that’d be stupid’. So, it’s about your self-esteem as well.

Participants also reported instances where they felt that other students did not fully understand or misinterpreted their different experiences of using discussion forums and other informal venues such as groups on text messaging services (e.g., WhatsApp). This can lead to feelings of loneliness in OSWD. Some participants revealed that they struggled disclosing their disability to others.

Participant 4: Will I tell them the truth [about my disability] or will I keep [it] a secret? It’s hard to know whether you should tell people that you’ve got a disability. ... When I revealed my [disability] to the [study] group, [they] thought that I was making up the fact that I couldn’t actually use WhatsApp on the phone. Are you saying I have to send you my medical records for you to believe me?

Participant 1: There is still some stigma surrounding identifying as disabled and I think it depends on the individual’s journey as to how comfortable they are about disclosing it. I wonder if maybe I would feel ... less lonely if I could just put it out there to my peers that I am disabled physically and mentally, so that it did not feel like I am hiding.

Participant 7: I think a really hard bit is not being able to explain what your disability is unless you’re actually working on a group project. If you suddenly announce you’re unwell and can’t do much, people think you’re making excuses.

Online learning can be socially challenging for all students due to lack of immediate communication and technological barriers (Elcicek et al., 2018; Song et al., 2019). However, these data extracts suggest that OSWDs’ understanding and experiences of loneliness, and social difficulties are often interwoven with their experiences of living with disabilities.

### Theme 3: activities, events and staff for informal socialisation are needed (RQ3: future support)

All participants identified areas for improvement in their online learning environment. Crucially, all participants stressed the importance of self-paced nature of online learning, consistent with online students without disabilities (Anderson, 2008).

Participant 8: I like studying online as it gives me flexibility. I usually inform my tutors when I am not active or engaging in the forum owing to my disability.

Interestingly, although participants reported difficulties with group activities and peer communication (Theme 2), they also noted that they would like to experience more collaborative activities, including online societies, group work and webinar style interactive sessions.

Participant 2: Depending on the nature of the club the social space can adapt, for example a photography club could be based on a platform focused on sharing photos.

Participant 7: I’d like small group seminars over Zoom or Skype to discuss ideas. That would help so much with learning and with meeting others on the course ... [instead of] just posting your work and reading the reply from your tutor.

Most of the participants reported wanting to experience social presence in their online learning environment. Social presence (i.e., students’ perception of ‘real people’ interacting with ‘real people’) is essential for authentic learning and can facilitate critical thinking (Garrison et al., 1999). Participants suggested having discussion forums for specific interests or hobbies (rather than academic purposes). Additionally, most participants wanted these activities to be separate from their university environment, to establish what might be considered a more authentic, informal social interaction. Participants also showed a need for ‘learner presence’, a presence that improves engagement where self-regulation optimises performance (Shea & Bidjerano, 2012) by suggesting simulating gaming environments. Participants also indicated the need for digital presence, suggesting being able to instantly message tutors or peers.

Participant 3: At my previous university and at my place of work you could instant message people within your course.

Participant 2: Perhaps an online way to interact that isn’t as static as a forum. There is more pressure on a post that remains there indefinitely that is also seen by course tutors. ... Other forms of online socialising, such as in online games.

Finally, our participants stressed the importance of having supportive tutors and technicians.

Participant 4: I couldn’t work [out] the thing that was being sent to me but the rest of the group expected that I would be able to do [it]. I just went quietly to one of the technicians, and they showed me how to do it. They didn’t make a fuss about it, they just did it [by showing] me how to do it–it’s about having that confidence I think.

Despite the relatively low expectations for socialisation in online learning (see Theme 1), OSWD still want to collaborate with others to establish authentic social interactions as opposed to formal ones. Their comments referred to some activities, venues and staff that facilitate informal (e.g., authentic, non-academic) socialisation among peers and staff, which would help them increase a sense of belongingness, and cope with a sense of loneliness in online learning. Table 2 summarised our RQs, themes, and examples of the participant excerpts.

**Table 2:** Research questions, themes and examples of participant excerpts

	Research question (RQ)	Theme	Illustrative quotes
1	What are the experiences of online learning and loneliness in OSWD? (Experience)	Self-paced study can reduce stigma but cause loneliness	“Online learning is great as it allows me as someone with a physical disability (wheelchair) to access higher education easily and comfortably … [But] when others are having discussions in the boards and I give my response, it feels like I am intruding and sometimes people will not respond to my posts.”
2	How do they make sense of their feelings of loneliness? (Understanding)	Loneliness and social difficulties relate to misunderstanding of disability	“Your disability might mean that you can’t sit for long. It might mean that you can’t read on the screen long. It might mean you can’t take in a whole lot of information quickly. You kind of get nearly left behind, and I think you’re at a greater disadvantage if you have a disability.”
3	What would help to reduce loneliness in online learning? (Future support)	Activities, events and staff for informal socialisation are needed	“I’d like small group seminars over Zoom or Skype to discuss ideas. That would help so much with learning and with meeting others on the course … [instead of] just posting your work and reading the reply from your tutor.”

## Discussion

This study aimed to evaluate the first-hand experience of online learning and loneliness among OSWD and how they make sense of their experience of loneliness, and discuss possible solutions for their loneliness. Data from semi-structured interviews with nine OSWD were analysed thematically, yielding three themes: i) Self-paced study can reduce stigma but cause loneliness; ii) Loneliness and social difficulties relate to misunderstanding of disability; and iii) Activities, events and staff for informal socialisation are needed. Each finding is discussed in turn. While noting our small sample size and the gender imbalance, the findings from the study are particularly relevant in a time where we are seeing an increase in online teaching and learning not just in higher education but across all levels of study, due to the COVID-19 pandemic (Kotera et al., 2020, 2021).

Self-paced study was regarded positively by our sample of OSWD allowing them to have enough time to digest information and circumvent stigma associated with the reduction of disability related features in online learning than the face-to-face settings. For example, students with physical disabilities in face-to-face settings are often worried if they will be able to access facilities and resources as students without disability would (Kotera et al., 2019). In online learning, concerns around whether their disability will be understood by others can be intensified as contacts with other students are often limited to their courses or other academic activities; there are less informal conversations taking place in online learning than face-to-face learning (Shu & Gu, 2018; Stodel et al., 2006). Casual conversations about their disabilities or worries associated with them, which often organically happen in face-to-face settings, can be helpful to address OSWD’s loneliness (Lin et al., 2015). Online learning institutions should systematically arrange opportunities for students to discuss these matters and foster inclusivity in educational communities.

In Theme 2, participants reported that loneliness and difficulties in their social aspect of online learning were uniquely related to misunderstanding of their disabilities. They noted high levels of worries that their disabilities and consequential behaviours may be misunderstood, which can make their online social life challenging. Relatedly participants noted a strong sense of isolation and alienation, two key components of loneliness (Rokach, 2015). Certain contextual factors in online learning can lead to an environment which does not fit the person’s abilities or capacities, contributing to disability. Indeed, an online learning environment with interactions which are not fully accessible can lead to an environment which does not meet the needs of OSWD, leading to a divide and thus exclusion (Hersh & Mouroutsou, 2019). Additionally, Theme 2 may highlight the broad definition and diverse degrees of ‘disability’, indicating that stigma associated with disabilities is still present somewhat in online learning. These variances in disability (i.e., types and degrees) can be narrowed down in face-to-face communication (e.g., some dyslexia students may have difficulties reading aloud, while others do not), though this is difficult to do in online learning, with limited opportunities to narrow down. Additional training about disabilities may be beneficial for online students (i) in order to create a safer learning environment where OSWD can learn without worries related to disability stigma, and (ii) considering a more inclusive learning environment for the large proportion of OSWD.

Our findings in Theme 3 highlighted that OSWD’s strong need for informal socialisation with others to cope with loneliness. Our participants emphasised that informal interactions with others are crucial for OSWD to cope with their loneliness, and suggested activities and events that can facilitate such nature of interactions, as well as supportive staff that would enjoy those types of interactions. Indeed, the importance of informal conversations, or chit-chat, has been highlighted in organisational psychology, regarding working from home (Methot et al., 2020)—particularly pertinent to the COVID-19 pandemic—, and our findings suggest similar positive effects can be applied to online education. Participants’ ideas including group work, more collaborative activities, more webinars and online societies would be worth evaluating, while considering data and attendee protection, and regulations. For example, using social media in education, including online education, has been actively discussed in the past years (Aydin, 2012; Prescott et al., 2013; Tang & Hew, 2017; Toker & Baturay, 2019). However, this may lead to procrastination and can overlook some teaching and student groups, as some universities or countries do not allow access to these tools, particularly relevant to online learning, where students enrol worldwide. Educators and course designers in online learning need to consider ways to facilitate informal socialisation within their context, evaluating the limitations and other characteristics of their environment. These may include instigating informal student-to-student engagement. One example is using group sets (e.g., groups of four to six students) which focus on socialising. Another example includes the use of socialising activities such as providing material in different formats where students are asked to identify associations to what is being shown (e.g., a picture showing different emotions provided with alternative text or long description as appropriate). Indeed, discussion prompts unrelated to course content has recently been suggested to combat loneliness in online learning (Kaufmann & Vallade, 2020).

Taken together, our findings suggest some ideas for future research that could lead to a number of practical implications. Firstly, exploring the prevalence of loneliness among OSWD, to explore determinants of loneliness, and to analyse the relationship of loneliness and academic performance would be worthwhile. Secondly, longitudinal research would be important for revealing naturalistic changes that take place throughout an online learning curriculum. Thirdly, engagement and impacts of disability education among online students may be valuable to be investigated. Lastly, the impacts of informal socialisation need to be assessed. For example, an empirical study to evaluate how informal socialisation among students can reduce the levels of loneliness among OSWD would help educators and course designers refine more effective interventions to counter loneliness in online learning.

While this study offers helpful insights into loneliness in OSWD, several limitations should be noted. First, institutional biases need to be considered as our sample was recruited from one UK-based university. Moreover, in addition to the small sample size, our sample consisted of only one participant identifying as male and all participants were European students. Their types of disabilities differed as well. A larger and more diverse sample would be warranted for future research to capture experiences from diverse backgrounds. This study also lacked a control group (i.e. students without disabilities), and in our recruitment advert, the study title explicitly stated our focus on loneliness; we may have only attracted participants with a specific disposition, who were willing to talk about their experiences of loneliness, and those unwilling to talk about their experiences of loneliness may not have been represented (including students who have a disability but have not declared).

## Conclusion

While the demands for online learning have increased among OSWD worldwide, investigations into their emotional wellbeing are limited. Previous research identified loneliness as a key detrimental factor for academic success in OSWD. This study aimed to appraise the experience and understanding of loneliness among OSWD, and discuss possible solutions for this problem. Our analysis revealed that the self-paced nature of online learning was regarded positively by OSWD, eliminating stigma associated with disabilities, but at the same time, they reported experiencing a sense of loneliness. Participants highlighted worries about being misunderstood in online learning where informal socialisation is less available than on-campus learning. Lastly, participants suggested ideas for reducing the sense of loneliness they experienced in online learning, with ideas such as group activities and online social groups for socialising with peers and staff. Our findings are intended to guide educators and course designers to initiate approaches to counter the sense of loneliness experienced by OSWD in online learning. Considering the rapidly increasing demand for online learning, urgency exists for measures to be evaluated and implemented.

## Supplementary Information

**Additional file 1.** Interview schedule.

## Data Availability

The datasets used and/or analysed during the current study are available from the corresponding author on reasonable request.

## Abbreviations

COVID-19: Coronavirus disease 2019
OSWD: Online students with disabilities
PG: Postgraduate
UG: Undergraduate
RQ: Research question
SWD: Students with disabilities
