# Supplementary material for: Loneliness in online students with disabilities: qualitative investigation for experience, understanding and solutions
Source: Int J Educ Technol High Educ. 2021 Dec 10;18(1):64. doi: 10.1186/s41239-021-00301-x (PMC8660147; doi:10.1186/s41239-021-00301-x)
Supplement: Supplementary file 1 — Additional file 1. Interview schedule. [file 41239_2021_301_MOESM1_ESM.docx]

**Loneliness in Online Students with Disabilities:**

**Qualitative Investigation for Experience, Understanding and Solutions**

*Additional Material 1. Interview schedule*

1. What kind of problems regarding socialising with others, if any, do you experience in relation to online learning?

2. In your opinion, what are some of the problems regarding socialisation that other students with disabilities may experience when learning online?

3. How often do you feel loneliness in online learning? (1 = Not at all, 7 = Always)

4. What are some of the ways you socialise with your peers online?

5. What do you think causes loneliness in online learning?

6. Do you think your disability causes loneliness in online learning?

7. Could you describe one or two incidents when you experienced loneliness in online learning?

8. Could you tell us what helps you to socialise or reduce loneliness in online learning?

9. Before your admission, did you think you would experience loneliness or difficulty socialising in online learning?

10. What do you hope for online learning in the future, that could counter loneliness for students with disabilities?
